# Carbon Nanotube Solar Cells

**DOI:** 10.1371/journal.pone.0037806

**Published:** 2012-05-24

**Authors:** Colin Klinger, Yogeshwari Patel, Henk W. Ch. Postma

**Affiliations:** Department of Physics and Astronomy, California State University Northridge, Northridge, California, United States of America; Texas A&M University, United States of America

## Abstract

We present proof-of-concept all-carbon solar cells. They are made of a photoactive side of predominantly semiconducting nanotubes for photoconversion and a counter electrode made of a natural mixture of carbon nanotubes or graphite, connected by a liquid electrolyte through a redox reaction. The cells do not require rare source materials such as In or Pt, nor high-grade semiconductor processing equipment, do not rely on dye for photoconversion and therefore do not bleach, and are easy to fabricate using a spray-paint technique. We observe that cells with a lower concentration of carbon nanotubes on the active semiconducting electrode perform better than cells with a higher concentration of nanotubes. This effect is contrary to the expectation that a larger number of nanotubes would lead to more photoconversion and therefore more power generation. We attribute this to the presence of metallic nanotubes that provide a short for photo-excited electrons, bypassing the load. We demonstrate optimization strategies that improve cell efficiency by orders of magnitude. Once it is possible to make semiconducting-only carbon nanotube films, that may provide the greatest efficiency improvement.

## Introduction

Solar cells have great potential as an alternative energy source because of the enormous amount of available energy and its distributed nature that may enable a distributed power generation grid [1]. However, for solar energy to be cost-effective on a utility scale, the price of purchase, installation, operation and maintenance over the lifetime of a solar panel per kWh generated must compare favorably to current power generation technology, which for fossil-fuel based generation is 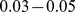
$/kWh [2]. Improvements are being made to solar cells to 1) increase the efficiency, and 2) lower the price. For instance, solar concentrators are being developed that focus solar light reflecting off a large mirror on a solar cell with a smaller surface area. Multi-junction devices are being developed that use junctions between materials with different band gaps to capture a greater number of photons and limit loss of excess photon energy when the excited high-energy electron relaxes to the Fermi level.

**Figure 1:**
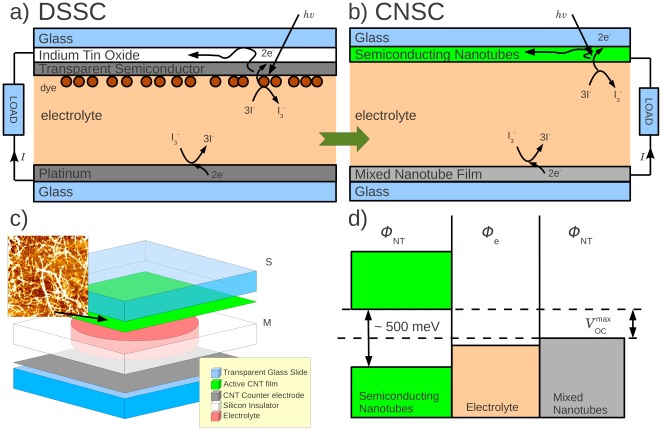
Carbon nanotube solar cells; comparison to Dye-Sensitized Solar Cells (DSSC), construction, and energeticts. a) DSSC. b) Carbon Nanotube Solar Cell, CNSC. c) Layout of a CNSC. The top and bottom glass slides (light blue) are covered in carbon nanotube films which are electrically connected by the iodide-triiodide electrolyte (light red) that is contained by the silicone separator (white). The top film (green) is the photoactive electrode, while the bottom electrode (grey) is the counter electrode. The inset is an Atomic Force Micrograph of the height of a 2×2 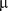
m section of a carbon nanotube film. d) Band diagram of the CNSC.

Gratzel cells [3], also known as Dye-Sensitized Solar Cells (DSSCs), offer a particularly interesting path to cost-effective solar power. By sacrificing some efficiency but offering a greater reduction in cost, the total price per kWh can be reduced considerably. While this initial argument for DSSCs is very compelling, it is worth noting that the current state-of-the art DSSCs have efficiencies that rival their solid-state counterparts [4]–[6]. Another advantage of DSSCs is that they operate well in low-light and overcast conditions. DSSCs typically consist of a transparent semiconducting film on conducting glass that functions as a photo-active electrode (figure 1a, top). A glass plate is coated with Pt and acts as the counter electrode (figure 1a, bottom). Light-sensitive dye molecules are adsorbed on a semiconducting material on another slide and the assembly is immersed in an electrolyte, typically iodide-triiodide (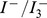). An incoming photon with energy 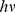 excites an electron from the dye into the conduction band of the semiconductor and it migrates to the bottom electrode. The electrolyte reduces the dye, creating triiodide (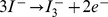). The electrons follow the external circuit through the load to the counter electrode. The triiodide migrates through the electrolyte to the Pt electrode and gets reduced, thereby completing the circuit. The transparent semiconductor is typically made of nanoporous TiO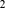. Using a nanoporous material significantly increases the surface area available for dye molecules but at the same time limits the electron migration rate. Different transparent semiconductors are being studied with higher mobility, such as nanowire-based electrodes [7], [8]. The liquid electrolyte is not very stable at the wide range of temperatures solar cells typically are exposed to, so high-mobility solids are being investigated as well [9], [10], culminating recently in a record 12% conversion efficiency [6]. Various dyes have been used in DSSCs, ranging from metal-free organic dyes [11] through highly efficient Ru-based organic dyes such as ‘N3 dye’ [12], [13] and ‘black dye’ [14]–[17] to engineered semiconductor quantum dots with a very high extinction coefficient [18]. C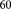 has been shown to work as a ‘dye’ as well [19], [20]. Carbon nanotubes (CNTs) [21], [22], offer a potentially cheaper and easier alternative to these materials. They are photo active, highly conductive, strong, and chemically inert. Carbon nanotubes can be synthesized in multiple ways such as chemical vapor deposition or laser ablation. The natural ratio of as-synthesized carbon nanotubes is 2/3 semiconducting to 1/3 metallic.

Here, we present proof-of-concept solar cells that are entirely made of carbon nanotubes, carbon-nanotube-based solar cells (CNSCs, figure 1b). They are a variation on the DSSC, and potentially offer many advantages beyond DSSCs. 1) **No Dye**. As these cells use semiconducting CNTs for photo conversion, they do not rely on dyes, which may bleach, severely limiting the useful life of DSSCs. 2) **No Pt**. Pt is often used as counter electrodes and their use in DSSCs represent an undesirable reliance on noble metals which may inhibit the use of DSSCs on a large, i.e. utility, scale. In addition, Pt has been reported to degrade due to the contact with the electrolyte [23]. Carbon nanotubes, in contrast, are chemically inert, and indeed show promising characteristics as counter electrodes [24]–[27]. 3) **No In**. As the carbon nanotube film itself is a transparent conductor, the use of a conducting coating made of, e.g. InSnO, is not required, eliminating the need for the exceedingly rare Indium. 4) The application of carbon nanotubes to the glass slides is a low temperature spray-coating process. In addition, these CNSCs multiply the advantages offered by DSSCs over single and multi-junction solar cells that require high-grade semiconductors and clean-room manufacturing. The use of low-grade materials and resulting projected significant reduction in cost of manufacturing potentially offsets the limited efficiency of these cells when relating the energy produced per dollar spent in manufacturing and installation.

In addition to CNT-only cells, we report on effiency improvement strategies, using different assembly techniques and using graphite (graphenium) counter electrodes. Graphite has no band gap, is extremely pliable, robust, and provides the ability to shrink the distance between it and the active semiconducting electrode. The cost, relative abundance, ease of introduction into the cell, and lack of need for spray deposition render graphite an attractive counter electrode material.

## Results

**Figure 2:**
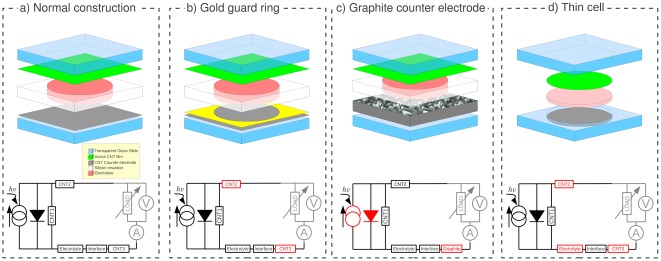
Layout of DSSCs and equivalent circuits. Both basic DSSCs (a) and tested optimization strategies are (b–d) are depicted. The circuit diagram is modeled after [41]. The alternative construction techniques lead to changes in the cell's electrical model, which are highlighted in red.

We present experimental demonstration of power generation obtained under ambient conditions at solar noon (see Methods section for details) of two types of cells. 1) *CNT-only cells*: cells are built with identical geometry but different CNT film compositions and thickness. This highlights how film composition affects cell performance (figure 2a). 2) *Optimized cells*: cells are built with the same CNT film thickness and composition, but with differences in construction techniques to isolate its role in cell efficiency (figure 2b–d).

### CNT-Only Cells (figure 1a)

**Figure 3:**
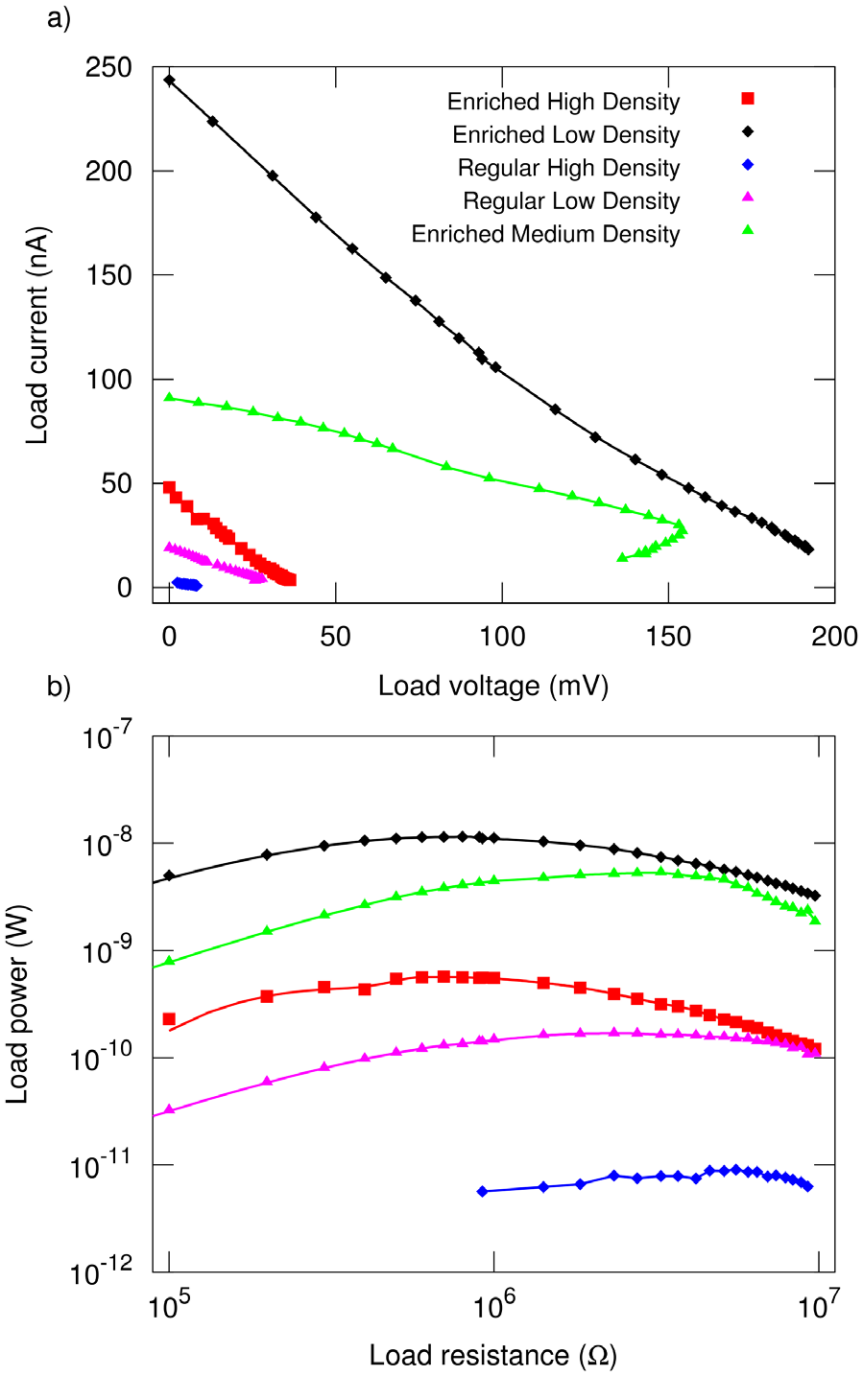
Electrical characteristics of the CNSCs. The extracted parameters are presented in table 2. a) 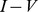 characteristics of the cells as indicated. b) Power delivered to the load for all cells as described in the legend for a).

The photocurrent 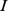 decreases linearly with increasing cell potential applied to the load 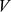 (figure 3a). We extract the open-circuit voltage 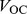 by extrapolating the 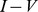 characteristic to 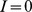 and the short-circuit current 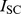 by extrapolating to 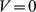 (table 1). Both 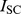 and 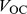 of the *enriched* mixture cells increase with decreasing CNT coverage of the semiconducting active electrode. Similarly, the high-density cell of the *regular* mixture of nanotubes has a lower 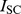 and 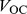 than the low-density cell. The power transfer curves (figure 3b) show a peak power transfer of 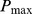 that occurs when the impedance of the load reaches 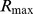. The low-density enriched as well as the low-density regular cells deliver more power to the load than their high-density counterparts. This is consistent with both 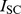 and 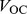 being larger.

**Table 1:** Parameters of CNSCs.

		Cell Type	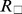	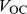	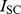	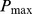	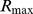	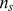	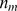
			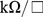	mV	nA	nW	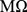	a.u.	a.u.
▪		Enriched High Density	3.4	43.6	47.0	0.57	0.71	521	58
♦	(black)	Enriched Low Density	62.7	208.5	243.4	11.50	0.82	121	13
♦	(blue)	Regular High Density	30.2	9.3	4.4	0.01	5.55	36	18
▴	(magenta)	Regular Low Density	50.3	21.4	19.0	0.17	2.45	29	15
▴	(green)	Enriched Medium Density	46.1	154.1	91.2	5.32	3.01	136	15

### Optimized cells (figure 2b–d)

**Figure 4:**
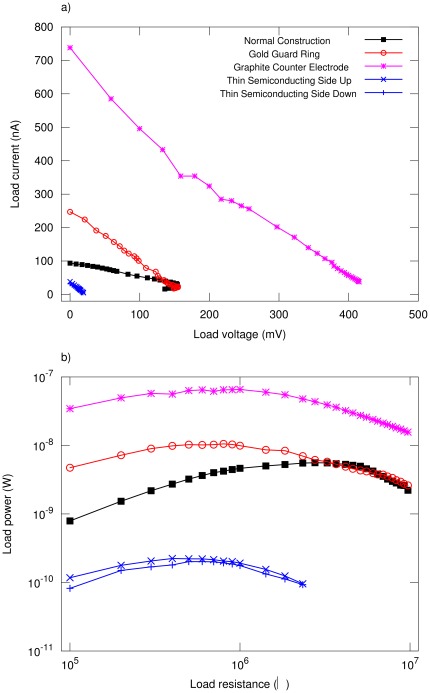
Optimization strategies for CNSCs. The extracted parameters are presented in table 1. a) 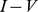 characteristics of the cells as indicated. b) Power delivered to the load.

We have studied cells with different construction techniques, using CNT electrodes from the same batch. Similar techniques were employed for data analysis as above (figure 4, table 2). The power transfer curves (figure 4b) show a peak power transfer 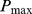 at 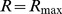. *Gold Guard Ring*. The presence of the gold guard ring increases 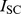 by a factor 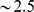, while 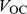 remains approximately constant. 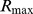 is lower by 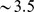 and 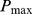 is 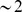 times greater. *Graphite Counter Electrode*. Both 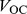 and 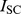 are greater than the normally constructed cell and 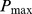 is 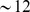 times greater. *Thin Cell*. When the enriched side is facing the incident solar radiation (“up”), the power is slightly larger than when the regular side is facing the incident radiation (“down”). Both 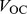 and 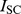 are lower by factors of 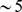 and 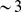, which in itself is undesirable. However, optimum power transfer occurs at a much lower resistance.

**Table 2:** Optimized CNSCs.

	Cell Type	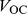	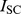	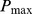	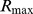
		mV	nA	nW	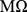
▪	Normal Construction	102.7	93.7	5.59	2.85
•	Gold Guard Ring	129.5	249.5	10.46	0.81
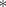	Graphite Counter Electrode	438.1	733.2	65.48	0.94
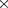	Thin Semiconducting Side Up	22.0	38.3	0.22	0.47
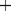	Thin Semiconducting Side Down	20.9	32.0	0.20	0.58

## Discussion

### Photocurrent generation and cell voltage

**Figure 5:**
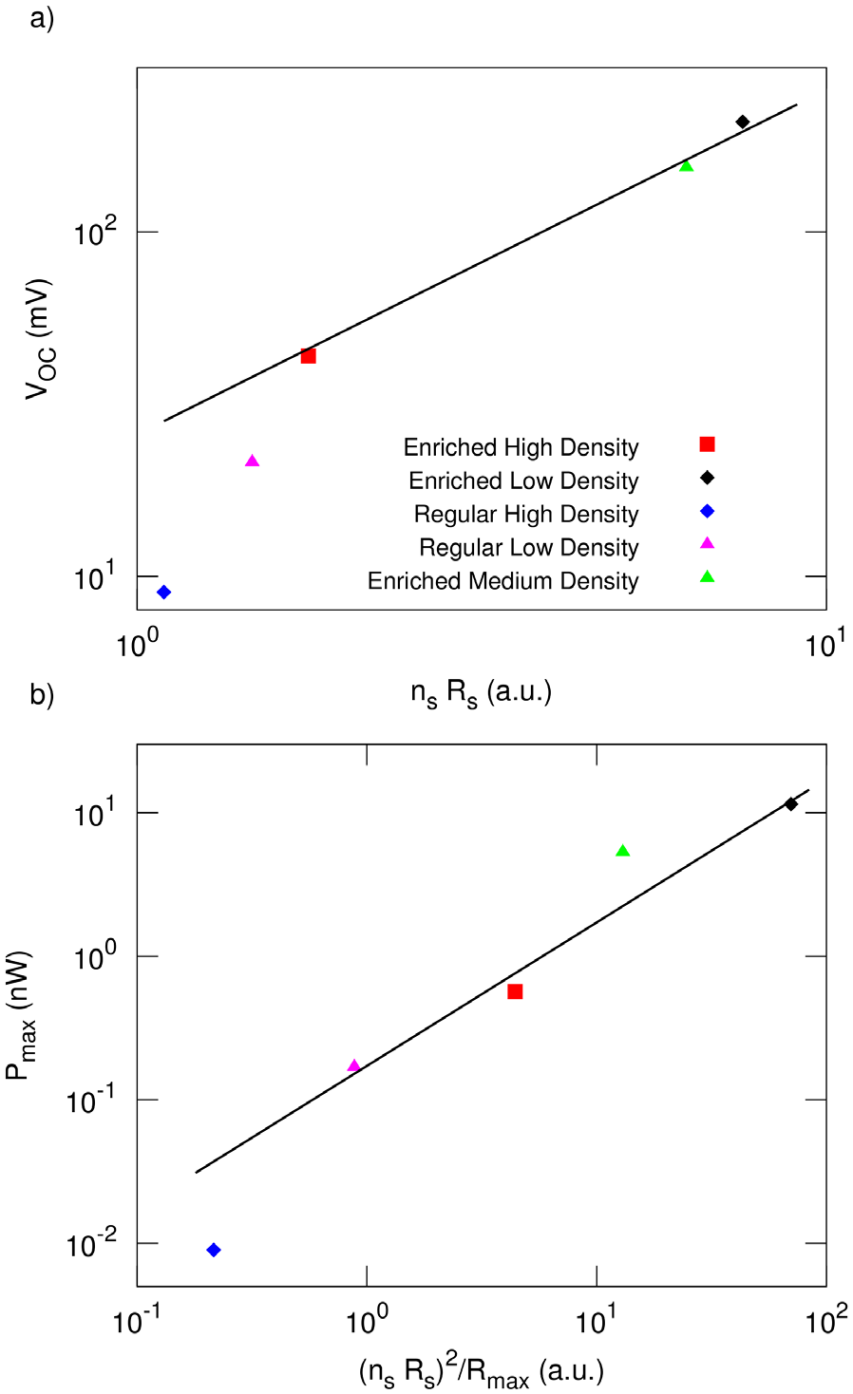
Scaling analysis of CNSC performance. The CNSCs characteristics are determined by the metallic and semiconducting carbon nanotube densities, with symbols corresponding to the cells as in the legend for figure 2. a) 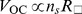. b) 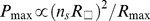.

The linear 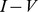 characteristic phenomenologically indicates the source is purely resistive, and maximum power occurs when the load and source impedance are equal. A figure of merit for solar cells that describes how close its 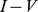 characteristic is to the ideal shape is the fill factor 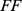 which is defined as the ratio of 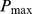 to the maximum power available with the corresponding ideal cell, 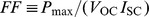. It ranges from 0 to 1, where 1 indicates an ideal cell. Ideal cells can supply a constant voltage independent on the load resistance up to the maximum current, when the voltage drops quickly to 0. Deviations from the ideal fill factor of 1 are usually due to parasitic resistances, such as shunt and series resistances. Shunt resistances affect behavior in the 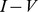 characteristic close to 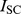, while series resistances affect performance close to 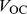. For our cell, 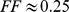. We argue that nanotube resistances 1–3 (figure 2) are responsible for this. Effectively, it means that the diode in the circuit diagram can be neglected. To estimate the number of nanotubes, we use the measured sheet resistance presented in table 1. Our CNT films are in the percolation limit [28], [29]. We can therefore use the scaling of sheet resistance with number of CNTs to extract the deposited volume of nanotube dispersion 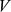, via
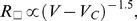
(1)where 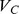 is the critical volume that determines the onset of conduction [28]. The volume can then be used to extract the surface density of metallic 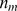 and semiconducting nanotubes 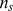. We assume that the metallic nanotubes dominate the sheet conductance, since their conductance 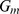 is much greater than semiconducting nanotubes 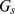. This assumption holds provided the conductance ratio 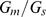 of metallic to semiconducting nanotubes exceeds the semiconducting to metallic abundance ratio 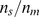. Single-molecule conductance studies of nanotubes indicate a conductance ratio of 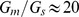, [30], [31] which supports our assumption that metallic nanotubes dominate the sheet conductance. We anticipate that for more enriched semiconducting films than studied here, a more detailed analysis will be required that takes into account the nanotube-nanotube contact resistance as well [32], [33]. The current-generating capacity of our cells is proportional to the number of semiconducting nanotubes 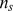. Combining both, the open-circuit condition corresponds to an ideal current source (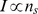) connected to CNT1 (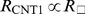) and the voltage developed across it will be
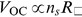
(2)and our data indeed approximately follows this scaling behavior (figure 5a). The outliers at low 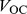 are CNT cells where both photoactive and counter electrode are coated with the same composition of carbon nanotubes. Both sides of the cell therefore create a photocurrent in opposite directions, but the light attenuation in the electrolyte breaks this symmetry and causes a directed current, albeit a smaller one and with a smaller voltage. The enriched cells further tilts the balance in favor of the photoactive side, leading to a 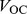 that is closer to that expected from the amount of nanotube material deposited on the active side alone.

Our cells have rather large output impedances and cannot maintain constant voltage over a larger range of load impedance. The cell output resistance can be reduced considerably by changing the aspect ratio of the cell, or connecting many cells in parallel. The output voltage can be held constant by a voltage-regulation circuit. However, there are many applications that do not require a low output impedance and would therefore work well with CNSCs, e.g. driving an LCD display or an E-Ink screen.

### Maximum Open Circuit Voltage

The band gap of semiconducting carbon nanotubes is related to the nanotube's diameter 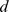 through
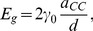
(3)where 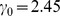
eV is the nearest-neighbour overlap integral and 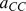 is the carbon-carbon distance [30], [34]–[36]. Note that the band gap of semiconducting nanotubes does not depend on the chiral angle. The band gap for a 1.5 nm nanotube, the average diameter of our nanotube material, therefore amounts to 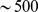
meV. The band diagram is drawn in figure 1d. The ‘work function’ of the electrolyte is 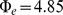
eV [37], while the work function for carbon nanotubes is 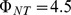
eV [36]. We therefore expect the maximum attainable open circuit voltage 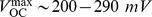, where the range indicates variations due to the diameter. We observe a maximum voltage of 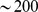
mV. This is expected as nanotubes with a slightly smaller band gap will ‘short out’ the effect of nanotubes with a slightly larger bandgap.

### Solar Power Generation

The maximum power delivered to the load is a function of 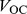 as well as the other resistances in the cell. The composite resistance of the cell is measured by determining at what value 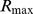 of 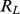 maximum power transfer occurs. Since we can model our cells as a voltage source with source voltage 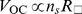, the maximum power available is expected to be 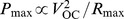, or
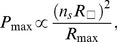
(4)and indeed the maximum power appears to follow this behavior approximately (figure 5b).

In summary, both 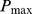 and 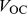 behave according to our model that describes the role of film's resistive properties on cell performance.

### Optimized Cell Designs

The *gold guard ring* causes 1) an increase in 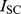, 2) an increase in 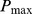, 3) a decrease in 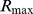, and 4) hardly any change in 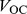. We believe this is due to a reduction of the resistance of the nanotube film in contact with the silicone insulator (CNT2 and CNT3, figure 2). The pressure of the insulator on the nanotube film as well as residual shear force during assembly may cause a perturbation of the nanotube film. In addition, the gold lowers the resistance of that part of the nanotube film. Both effects combined act to lower 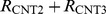. As the open-circuit voltage is independent of 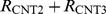, the open-circuit voltage should be unaffected by this improvement in design, and indeed we observe 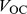 (Normal) 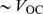 (Gold Guard Ring). The reduction of 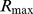 is also explained by the reduction of 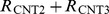, and that, in turn, explains the increase of both 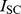 and 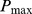.

The employment of a *graphite counter electrode* instead of a carbon-nanotube counter electrode not only improves 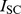, but also 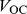. The increase in 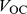 is due to the use of graphite instead of carbon nanotubes. The increase in 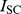 is due to the lower sheet resistance of the graphite as compared to a carbon-nanotube film. The magnitude of the improvement is similar to that accomplished with the gold guard ring improvement, as the maximum power transfer occurs at approximately the same load resistance [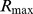 (Gold Guard Ring) 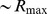 (Graphite Counter Electrode)]. Graphite is preferred over gold, naturally, to reduce cell cost. Both effects combine to increase the power output of the cell by a factor of 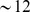.

The reduction of the distance between active and counter electrode for the *thin cells* can reasonably be expected to lower the resistance of the electrolyte. In addition, as these cells were constructed without a silicone separator, we observe that the maximum power transfer occurs at a much lower resistance. This is due to the absence of the disruptive effect of the silicone separator, which role was elucidated by the study of the gold guard ring device above. The reduction of the electrolyte chamber thickness also has an adverse effect. The electrolyte absorbs less solar radiation than with thicker devices. Therefore, both the enriched (photo active) side, as well as the regular mixture side create a photo current. However, both sources have opposite polarities, causing the effective open-circuit voltage to be reduced as we indeed observe.

### Efficiency

The average solar flux during testing was 770 W/m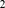, and the greatest solar power generation was attained with the graphite counter electrode and enriched medium-density CNT active electrode. The efficiency of that cell was 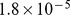. Compared to the all-CNT construction, an improvement of more than a factor 10 was attained. If a cell were constructed with the graphite counter electrode and the low-concentration CNT enriched active electrode, an increase of power by a factor 2 is anticipated. This can be deduced by comparison of the medium density enriched cell to the low density enriched cells with the regular construction. As the graphite counter electrode lowered the output resistance by a factor 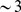, the power output may be larger by a factor 3 as well. Further improvements may be obtained by changing the aspect ratio of the solar cell. In the design reported here, we used effectively square films. Changing the cell design by making the cells wider, will lower the resistance further. An aspect ratio of 10 can then reduce the film resistance by a factor of 10, causing a reduction of 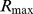, which will improve 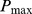. Our thin cell results indicate that the largest resistance is due to the nanotube film, we therefore believe the efficiency increase with this improvement may be as large as 10-fold. We believe the greatest efficiency increase may be obtained by using CNT source material with a greater fraction of semiconducting nanotubes. The films we used had 90% semiconducting nanotubes and 10% metallic nanotubes. As we argued above, the semiconducting nanotubes provide the photo-generated current, but the metallic nanotubes short the load. If one were to use 99% semiconducting films, the amount of nanotubes could be increased by a factor 10, while still maintaining the same number of metallic nanotubes. As metallic nanotubes are more conductive than semiconducting nanotubes, we assume that the number of semiconducting nanotubes can be increased by this factor 10 without affecting 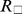 and 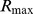. Future generation cells can then reasonably be expected to deliver 100 times more power, due to the increase of 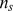 by a factor 10 (equation 4). However, at a certain abundance factor of semiconducting to metallic nanotubes, this argument will not hold any longer. Combining all of these improvements may lead to an efficiency of 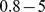
%, where the lower bound is a conservative estimate that every improvement will only contribute half we argued above. We hope the studies reported here will motivate further development of methods to create highly-enriched semiconducting CNT source material cost effectively at a large scale.

## Materials and Methods

### Cell Construction

The *enriched* CNSCs (figure 1) consist of a transparent glass slide covered with an enriched mixture of 90% semiconducting and 10% metallic nanotubes (IsoNanotubes-S 90% Powder, Nano Integris Inc.). These nanotubes have a diameter of 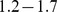
nm and a length of 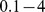 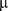
m. Below this is a silicone insulator with a hole filled with electrolyte (iodide-triiodide, Solaronix). The electrolyte is in contact with both carbon nanotube films and acts to reduce the photo-active side as well as close the electrical circuit at the counter electrode. At the bottom is a glass slide covered with a regular mixture of 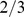 semiconducting and 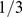 metallic nanotubes that acts as a counter electrode (Unidym, lot PO-325, formerly Carbon Nanotechnologies Inc.). These nanotubes have a diameter of 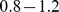
nm and a length of 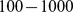
nm. Dispersion of carbon nanotubes were made by ultrasonic agitation in 1,2-dichloroethane for 1 h for the regular mixture and 4 h for the enriched mixture. The dispersion was spray painted with an air brush onto glass substrates in a vented cylindrical enclosure. The slides were rotated while spraying to obtain uniform coverage. Subsequently, the glass slides were heated on a hot plate to evaporate any residual solvent. The resulting film is similar to the well-known bucky paper and it has metallic properties [38]–[40]. The final solar cell has an exposed surface area of 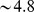
mm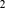 with a distance of 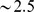
mm between the electrodes. The glass slides are 1 mm thick and did not have a conducting coating prior to carbon nanotube application.

### Gold guard ring (figure 2b)

A mask the size of the opening containing the electrolyte was placed onto the glass slide after CNT deposition, followed by Au deposition. This procedure prevents degration of the metal due to the electrolyte contact.

### Graphite cell (figure 2c)

A cell using graphite (graphenium) as the counter electrode was created. The graphite cell counter electrode construction consists of the same steps for deposition of semiconducting CNTs. A PDMS (Slygard 184 Silicone Elastomer, Dow Corning Corp.) plastic mold with a circular depression was created to house pieces of graphite of different heights. A wire is placed through the PDMS at the height of the bottom of the depression. After graphite deposition the PDMS was filled with electrolyte and the active semiconducting electrode was placed on top of the cell.

### Thin Cell (figure 2d)

A cell with a separation of about 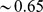
mm between the active 90% semiconducting electrode and a regular CNT counter electrode was created. A piece of 1 mm thick glass was locally machined to create a central depression with a connection to a ramped section. The glass was cleaned and masked in the non-machines areas. The glass was sprayed with the 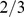 semiconducting and 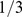 metallic nanotubes mixture. A second piece of glass was masked with the same pattern as the machined glass piece and sprayed with 90% semiconducting CNTs. The two nanotube electrodes were connected to external electrodes and the cell was filled with electrolyte and sealed with liquid silicone sealant. The liquid silicone was allowed to dry and harden creating a seal. The thin cell has an exposed surface area of 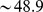
mm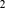 with a distance of 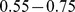
mm between the electrodes.

### CNT film preparation and characterization

The spray-painted CNT slides were imaged with an Atomic Force Microscope (Dual-Scan AFM, Pacific Nanotechnology, USA) to determine the coverage (figure 1). In addition, a probe station was used to measure the sheet resistance 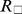 (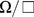) of the CNT films, by analyzing the distance dependence of the two-terminal resistance 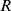 as a function of probe separation 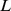 on a semilog scale and performing a least-squares fit to
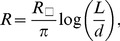
(5)where 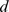 is the probe tip diameter. The counter electrodes used in this study were all obtained from the same batch in order to ensure uniformity and their sheet resistance was 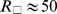
k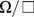.

### Solar Power Generation measurements

The assembled cells were connected to a load resistor 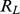 that was varied from 0 to 10 M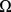 through a current amplifier and the voltage 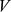 across and current 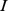 through it are measured as a function of 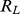 (figure 2). The cells were pointed straight at the sun and were measured in Northridge, CA (visibility: 10 miles, Latitude 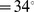
N) at solar noon from Dec 2010 through April 2011. The sun's altitude 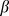 was between 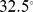 and 65.7, yielding an air mass of 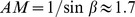. The average solar flux was 770 W/m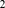. To minimize the effect of variability in solar conditions and cell assembly, devices were fabricated with large variations in carbon nanotube concentrations to highlight its effect on cell performance.

CNTs were created in batch operations, providing the ability to test various parameters and the resistances of electrodes used in experiments.
